# Foraging benefits of winning intergroup encounters in colobus monkeys

**DOI:** 10.1007/s10329-026-01272-1

**Published:** 2026-06-18

**Authors:** Eva C. Wikberg, Sara Lucci, Emily Glotfelty, Fernando Campos, Pascale Sicotte

**Affiliations:** 1 Department of Anthropology, University of Texas at San Antonio, MH 4.03.38, UTSA, One UTSA Circle, San Antonio, TX 78249 USA; 2 Department of Anthropology and Archaeology, University of Calgary, Calgary, AB Canada; 3 Department of Biology, Concordia University, Montreal, QC Canada

**Keywords:** Between-group contest, Feeding competition, Differential access to resources

## Abstract

Winners of aggressive intergroup encounters are often assumed to benefit from increased access to resources, but few studies have measured whether there is differential access to resources based on the intergroup outcome. To investigate whether winning intergroup encounters is associated with increased access to food, we used 13 months of data on intergroup dynamics, feeding by 61 females, and the phenology and spatial distributions of food trees from the home ranges of 8 groups of white-thighed black-and-white colobus (*Colobus vellerosus*) at Boabeng-Fiema, Ghana. Contested trees were important food species more often than expected based on the occurrence of these species in the study area. Contested trees had young leaves, fruits, and seeds more often than expected based on the presence of these plant parts on trees monitored via monthly phenology surveys. Focal females were more likely to feed on the contested tree than other trees they were in during and immediately after the intergroup encounter. Based on group scan data collected throughout the day of an intergroup encounter, the likelihood of females feeding on any food species before and during the intergroup encounter was similar regardless of the outcome. The likelihood of feeding was higher after the intergroup encounter if the group had won compared to if the group had lost. These findings indicate that groups compete over access to important food resources and winners benefit from increased access to food. Future studies should investigate whether winning intergroup encounters is associated with long-term benefits such as increased reproductive success.

## Introduction

Intergroup encounters are characterized by aggressive behaviors in a wide range of taxa (Boydston et al. 2001; Bruintjes et al. 2016; Cheney 1987; Dyble et al. 2019; Hale et al. 2003; Holldobler 1981; Majolo et al. 2020; Radford 2008). During these aggressive encounters, individuals often compete against individuals in other groups over access to limiting resources such as home ranges or territories (Adams 1990; Isbell et al. 2021; Lemoine et al. 2020a, b; Packer et al. 1990), mates (Cant et al. 2002; Perry 1996; Saito et al. 1998; Schoof and Jack 2013; Sicotte 1993; Sicotte and MacIntosh 2004; Yi et al. 2020), and food resources, water holes, or sleeping sites (Golabek et al. 2012; Harris 2006; Korstjens et al. 2005; Markham et al. 2012). The outcome of an intergroup encounter can have important implications for male and female reproductive success if the benefits of winning access to these limiting resources outweigh the energetic costs associated with participating in intergroup aggression (Cheney and Seyfarth 1987; Lemoine et al. 2020a, b). The loser of an intergroup encounter may incur additional costs such as increased travel distances and reduced use of the area after the intergroup encounter (Crofoot 2013; Dyble et al. 2019; Koch et al. 2016; Markham et al. 2012).

The outcome of an intergroup encounter is not only determined by the competitive ability of the opponents, but also by the perceived value of the contested resource, which influences the motivation to fight for access to that resource (Maynard Smith and Parker 1976; Parker 1974). Food abundance and competition predict intergroup aggression in many species (García et al. 2022; Golabek et al. 2012; Harrison 1983; Korstjens et al. 2005; Lewis et al. 2020; Pal 2015; Thompson et al. 2017). For example, intergroup aggression is more likely to occur in feeding contexts in free-ranging female dogs (*Canis familiaris*) (Pal 2015), with increased competition in banded mongooses (*Mungos mungo*) (Thompson et al. 2017), and when foraging on seeds (a high-value resource) in female western black-and-white colobus (*Colobus polykomos polykomos*) (Korstjens et al. 2005).

The winner of the intergroup encounter is assumed to benefit from increased access to the contested resource; however, relatively few studies have investigated empirically whether winners benefit from an immediate increase in access to food. In a study that investigated this, guerezas (*Colobus guereza*) in the winning group took over the contested tree in approximately 70% of cases, and the likelihood of this occurring increased if the tree was of an important food species (Harris 2006). Several studies have investigated whether winning an intergroup encounter or a group’s competitive ability affects movement behaviors, home range size, and/or home range quality (Crofoot 2013; Dyble et al. 2019; Koch et al. 2016; Lemoine et al. 2020a, b; Markham et al. 2012; Scarry 2013), which could increase access to food over a longer timescale. For example, black capuchin (*Sapajus nigritus*) groups with a higher number of males were more likely to win intergroup encounters, and have more exclusive use of their home ranges that provide them with greater per capita food abundance (Scarry 2013).

Other studies point to a lack of significant differences between winners and losers in access to contested resources. In some green monkey (*Cercopithecus sabaeus*) intergroup encounters, individuals in one group aggressively displaced an opposing group without meeting any resistance despite the opposing group being able to win intergroup encounters against the former at other locations (Harrison 1983). These one-sided intergroup encounters occurred over food resources that were rare within the winning group’s home range while the losing group’s home range contained many trees of this species. Because they had access to the same tree species in other parts of their home range, the individuals in the losing group may not have incurred significant costs. In feral horses (*Equus ferus caballus*), the stallion in the band that arrives first at a water hole defends access to it until his band has finished drinking, after which another band can take it over (Stevens 1988). Thus, the band is not giving up access to this limited resource until its relative value has decreased. Similarly, red-tailed monkeys (*Cercopithecus ascanius*) that have already fed to satiation on a food resource are more likely to cede that resource to an opposing group whose individuals have lower energetic balance and greater motivation to feed (Brown et al. 2022). In this study, red-tailed monkeys improved their energy balance after winning intergroup encounters, but they started in a worse condition than individuals in the losing group. Red-tailed monkeys in the losing group do not pay short-term costs in the form of increased travel distances or reductions in energy balance after the intergroup encounter.

In the population of white-thighed (or ursine) black-and-white colobus (*Colobus vellerosus*) at Boabeng-Fiema, males are aggressive during the majority of intergroup and extra-male encounters to defend access to mates, although they may also defend infants and food (Sicotte and MacIntosh 2004). However, female intergroup aggression has increased over time with increased population density and is predicted by seasonal availability of high-quality food items (Arseneau-Robar et al. 2024). These findings indicate that females may defend access to food when high-quality items are available. During the rainy season, the colobus feed mostly on mature leaves, which may be a fallback food source when more nutritious food items are unavailable (Saj and Sicotte 2007a). Coparticipation in intergroup aggression is more likely in smaller groups (Wikberg et al. 2022a, b), and smaller groups and/or groups with high-quality males are more likely to win intergroup encounters (Teichroeb and Sicotte 2018; Wikberg et al., In prep.). Although these previous studies have analyzed predictors of intergroup encounters and intergroup aggression, it remains unclear whether female resource defense is important for increasing access to food and whether winners have better access to food than losers. First, male colobus are important in intergroup aggression with the main function to defend access to mates (Sicotte and MacIntosh 2004). Second, it may be possible for colobus in losing groups to relocate and resume feeding relatively quickly, because home ranges are small (Teichroeb and Sicotte 2009) and groups do not chase each other over long distances (Wikberg, personal observation). Indeed, losers do not always incur significant costs even when groups compete over access to food patches in other study populations (Brown et al. 2022; Harrison 1983; Stevens 1988).

This study focused on intergroup encounters when females defended access to resources to investigate whether the outcome of these intergroup encounters were associated with feeding-related costs and benefits. We predict that contested trees will be of species that make up a large percentage of the annual diet, and that the contested trees will have high-quality food items (young leaves, flowers, fruits, or seeds). If the contested trees are particularly important for feeding, we also predict females to spend more time feeding in the contested trees than in other trees they are in around the time of the intergroup encounter. If winners benefit from increased access to food resources overall, we expect individuals in the winning group to be more likely to forage on any type of food species. In contrast, losers may not incur significant foraging costs if they can relocate to another food patch relatively quickly or if they have already fed to satiation. In the former case, there would not be a significant difference in feeding after the intergroup encounter between individuals in losing and winning groups. In the latter case, individuals in the losing group are expected to have higher likelihood of feeding before the intergroup encounter compared to individuals in winning groups.

## Methods

### Study population

The population of white-thighed black-and-white colobus at Boabeng-Fiema in central Ghana (7°43’N and 1°42’W) occupies a 1.92km^2^ fragment of semi-deciduous forest (Hall and Swaine 1981). The population has increased over time due to improved protection, and the forests by the villages of Boabeng and Fiema contained around 300 individuals in 19 groups at the time of this study (Fargey 1992; Holmes 2011; Kankam et al. 2010, 2023; Saj et al. 2005; Wikberg et al. 2023; Wong and Sicotte 2006). All males disperse while only about half of the females disperse from their natal group (Sicotte et al. 2017; Teichroeb et al. 2009, 2011; Wikberg et al. 2012). Males form weak affiliative relationships with other males and rarely cooperate with each other (Teichroeb et al. 2014; Wikberg et al. 2012). Females form stronger social bonds than males in most groups, and females’ social networks are influenced by the presence of young infants, female immigration status, and kinship, but not by dominance rank (Wikberg et al. 2012, 2013, 2014a, b, 2015). Females sometimes form coalitions against group members or extra-group members (Wikberg et al. 2014a, b). In a previous analysis, at least one group member showed aggressive displays, chases, or contact aggression in 72% of the intergroup encounters the group was involved in (Wikberg et al. 2022a, b), and intergroup aggression was more common for males than females in most groups (Arseneau-Robar et al. 2024; Sicotte and MacIntosh 2004; Teichroeb and Sicotte 2018; Wikberg et al. 2020, 2022a, b).

### Data collection

We collected behavioral and ecological data from May 2008 to June 2009 as part of a study to investigate the links between intergroup contest, dyadic bonds and cooperation, and access to food. For one group, data collection began in September 2008, when we had learned to recognize all individuals. The study groups had neighboring home ranges with two to five of the other study groups. The eight study groups each consisted of 1–9 adult and subadult males and 4–13 adult and subadult females. Each morning, we contacted one “focal” group and stayed with the group until the end of the day or until we lost contact with the group. We contacted the groups in a set order to make sure we had an even number of focal hours per group. If the intended focal group could not be located or was in an area where it was impossible to do observations due to dense foliage and lack of trails, we would instead observe a group that could be located. The focal group was our main observation group from which we collected baseline behavioral data throughout the day. We would often collect *ad libitum* data from other groups during intergroup encounters. To reduce disturbance to the habitat and the primates, observers stayed on existing trails. Observers maintained a distance of at least 10 m, but most often observed the primates when they were in trees at a distance of 20–40 m. If the primates went down low in dense vegetation and were no longer visible from the trails, the observers would pause data collection.

To determine the focal group’s movement pattern and the outcome of intergroup encounters, we noted its location once an hour using a map with trails and all large trees (with a diameter at breast height (DBH) of 40 cm or more) identified and mapped. We defined an intergroup encounter as when two or more groups were within 50 m of each other (Oates 1977; Sicotte and MacIntosh 2004). During intergroup encounters, we recorded the location of the groups, social behaviors directed to individuals in the opposing group, and the identity of interactants *ad libitum* (Altmann 1974). If possible, we also recorded interactants from the non-focal groups. We defined aggressive intergroup participation as chasing of or contact fighting with extra-group members, or performing aggressive stiff-leg or jump displays (Oates 1977). We coded the intergroup encounter outcome as “win” if the focal group displaced the opposing group by taking over the location or tree that the opposing group had occupied, “lost” if the opposing group displaced the focal group, and “draw” if none of the groups took over the other group’s location or tree. Whenever possible, we also recorded the species and phenology of trees that were contested during intergroup encounters.

To investigate whether the contested trees were more important for feeding in association with the intergroup encounter than other trees, we used focal follows during which the focal animal’s behaviors and tree usage were recorded. We collected 10-minute focal follows (Altmann 1974) of adult and subadult females throughout the day. If the focal female went out of view before the end of the 10 min, we retained the focal follow in our analysis if the female was in view for at least 5 consecutive minutes. We had an initial order of focal females, which we used to choose our next focal individual. If it was not possible to find the next female on the list or to collect data on that female due to low visibility, we moved to the next female on the list. If we had an uneven number of focal follows per female, we prioritized the female with the lowest number of focal follows. We only collected one focal follow per female each hour, except in the smallest study groups with only five females, in which it was possible to collect focal follows of all focal females and restart the cycle within one hour. During point samples every 2.5 min during the focal follows, we recorded the focal female’s activity (forage, locomote, rest, self-directed behaviors, social behaviors, vigilant, and other). We also recorded which tree the focal animal was in.

To analyze whether feeding on any species during the day of an intergroup encounter varied with the outcome of the intergroup encounter, we collected group scans (Altmann 1974) once an hour of group members of all sex-age classes. We recorded the individual’s activity (forage, locomote, rest, self-directed behaviors, social behaviors, vigilant, other), plant part and species if foraging, and its proximity to others.

To assess the availability of high-quality food items on trees in the study area, we used data from phenology surveys. Once a month, we visited the same phenology trees that included up to five trees of each food species from each group’s home range to record whether each plant part (flowers, fruits/seeds, young leaves, and mature leaves) was present (Saj and Sicotte 2007a).

### Data analyses

We only included focal group intergroup encounters with female aggression and at least one contested tree in our analyses. Contested trees were those for which individuals in one group attempted or succeeded in displacing individuals from another group. We excluded aggressive intergroup encounters in which only males participated, because a main function of male aggression is to defend access to mates (Sicotte and MacIntosh 2004). We used a chi-square test to investigate whether the observed numbers of intergroup encounters over at least one contested tree of an important food species (i.e., made up 5% or more of a group’s annual diet based on feeding point samples from focal follows) vs. intergroup encounters over contested trees that were nonimportant food species (Saj and Sicotte 2007a) differed from expected numbers. Other trees that individuals were in during an aggressive intergroup encounter, but that did not meet our definition of a contested tree, were not included in this analysis. We only included contested trees in our analysis if they were individually identified and mapped, as we were comparing their characteristics with those of all mapped trees (i.e., all trees in the study area with a DBH greater than 40 cm). We calculated the expected numbers of contested trees of important and nonimportant food species by multiplying the total number of contested mapped trees by the proportion of all mapped trees that were important (251/722) or nonimportant food species (471/722), respectively. We also used a chi-square test to investigate whether the observed numbers of contested trees with or without high-quality food items (i.e., a binary variable indicating the presence or absence of young leaves, flowers, fruits, or seeds) differed from expected numbers. The expected numbers were calculated by multiplying the total number of contested trees with associated phenology data by the proportion of monthly phenology tree records with or without high-quality food items, respectively.

To examine whether contested trees were more important than other trees for feeding in intergroup contexts, we used focal follow point samples during which the focal animal’s behavior and tree usage were recorded. We only included focal follows that were collected during or within one hour after an intergroup encounter where we had identified a contested tree. We used the one-hour time window after the intergroup ended because we wanted to investigate the potential immediate benefits of winning access to the contested tree. We had collected female focal follows during and within one hour of the end for 76 of the 96 intergroup encounters with female aggression over contested trees. We were unable to collect focal data during the other 20 intergroup encounters due challenging observational conditions and limited visibility. If the focal female switched trees during the follow, each focal follow segment in the same tree contributed one data point to the analysis (*N* = 625 focal follow – tree combinations). We set up a generalized linear mixed model with a beta-binomial distribution to investigate whether the number of point samples spent feeding to the number not spent feeding during focal follows during and within one hour after the encounter ended was predicted by: (1) whether the focal female was in a tree that had been contested or not during the intergroup encounter; (2) whether it was dry season (November-April with low rainfall and high young leaf availability) or rainy season (May-October with more rainfall and low young leaf availability) (Saj and Sicotte 2007b); and (3) adult and subadult group size. Although the latter variables were not part of the predictions we set up to test, we included them because season and group size predict feeding in non-intergroup encounter contexts (Saj and Sicotte 2007b; Teichroeb et al. 2003; Teichroeb and Sicotte 2009). We included group and focal identities as random effects in this model.

To analyze whether feeding on any species during the day of an intergroup encounter varied with the outcome of the intergroup encounter, we used 637 group scans of adult and subadult females collected during days when the group was only involved in one intergroup encounter with one other study group. We created a GLMM with the outcome variable of whether the scanned female was feeding (yes/no). Because there were many records without feeding, we used a cloglog link function. We included an interaction effect between the outcome of the intergroup encounter (win, draw, loss) and the intergroup encounter status (before, during, or after the intergroup encounter). We also added the fixed effects season (rainy or dry), group size, and the interaction between group size and outcome. We included group ID and observation session ID (i.e., a continuous block of observation of the same group) as random effects.

In the GLMMs, we used z-transformations to standardize all continuous fixed effects (Schielzeth 2010). We performed all our analyses in R with the packages glmmTMB (Brooks et al. 2017) and multcomp (Hothorn et al. 2014). We used the R packages DHARMa (Hartig 2021) and performance (Lüdecke et al. 2021) to assess model fit and calculate VIF. The fixed effects showed low collinearity (VIF < 1.14).

## Results

The focal group was involved in 232 intergroup encounters (i.e., the focal group was within 50 m of another group). We were able to collect behavioral data during 173 intergroup encounters, and females showed aggression in 119 of these intergroup encounters. In 23 of the 119 intergroup encounters with female aggression, we could not identify a specific tree from which individuals in one group attempted or succeeded in displacing individuals from the other group. Individuals attempted to or successfully displaced individuals in the opposing group from specific trees in 96 of 119 intergroup encounters with female aggression. These 96 intergroup encounters make up the data set used for analyses. During each of these 96 intergroup encounters with female intergroup aggression over access to trees, 1–8 females and 0–4 males from the focal group directed aggression or aggressive display behaviors to the opposing group. The duration of the intergroup encounter ranged from 16 min to almost 9 h (mean + SD = 2.50 + 1.76 h, median = 1.95 h).

### Characteristics of contested trees

During each of these 96 intergroup encounters, 1 to 6 mapped trees were contested. There were 89 unique contested trees of 31 different species, and each of the trees was contested during 1 to 4 intergroup encounters. The contested trees were important food species (≥ 5% of a group’s annual diet) in 83% (80/96) of intergroup encounters, were less-important food species (< 5% of annual diet) in 14% (13/96) of intergroup encounters, and were not a food species in 3% (3/96) of intergroup encounters. In contrast to these patterns, only 32% of all 1,938 mapped trees were classified as an important food species. There was a significant difference in the observed versus expected numbers of intergroup encounters with contested trees that were important food species (Table 1).

Of the 40 contested food trees for which we collected phenology data on the day of the intergroup encounter, all but one tree had high-quality food items: 19/40 trees had young leaves, 18/40 trees had young leaves and fruits/seeds, 2/40 trees had fruits/seeds. The one contested tree with no high-quality food items had mature leaves only. We recorded the presence of young leaves in 26% and fruits or seeds in 5% of the 2,631 monthly phenology records. Accordingly, the observed numbers of contested trees with and without these food items differed significantly from a null expectation (Table 1).

**Table 1:** Observed and expected numbers of intergroup encounters (IGE) over trees that were important food species and observed and expected numbers of contested trees with young leaves, fruits or seeds

Tree characteristics	Observed number	Expected number	χ^2^	*p*-value
IGE with important food species	80	31	49.20	< 0.001
IGE without important food species	16	65		
Tree with young leaves	39	11	38.88	< 0.001
Tree without young leaves	1	29		
Tree with fruits or seeds	20	2	18.12	< 0.001
Tree without fruits or seeds	20	38		

### Feeding on the contested tree

We had collected female focal follows during and within one hour of the end for 76 of the 96 intergroup encounters with female aggression over contested trees (*N* = 625 focal follow – tree combinations). Females were feeding during 23% of point samples when they were in the contested tree and 17% of point samples in other trees. Other common activities were rest (42% and 45% of point samples in contested and other trees respectively) and vigilance (23% versus 25% of point samples in contested versus other trees). All other activities made up 12–13% of the point samples in contested trees versus other trees (1–4% affiliative, display, locomote, play, self-directed behaviors, and other behaviors).

**Fig. 1:**
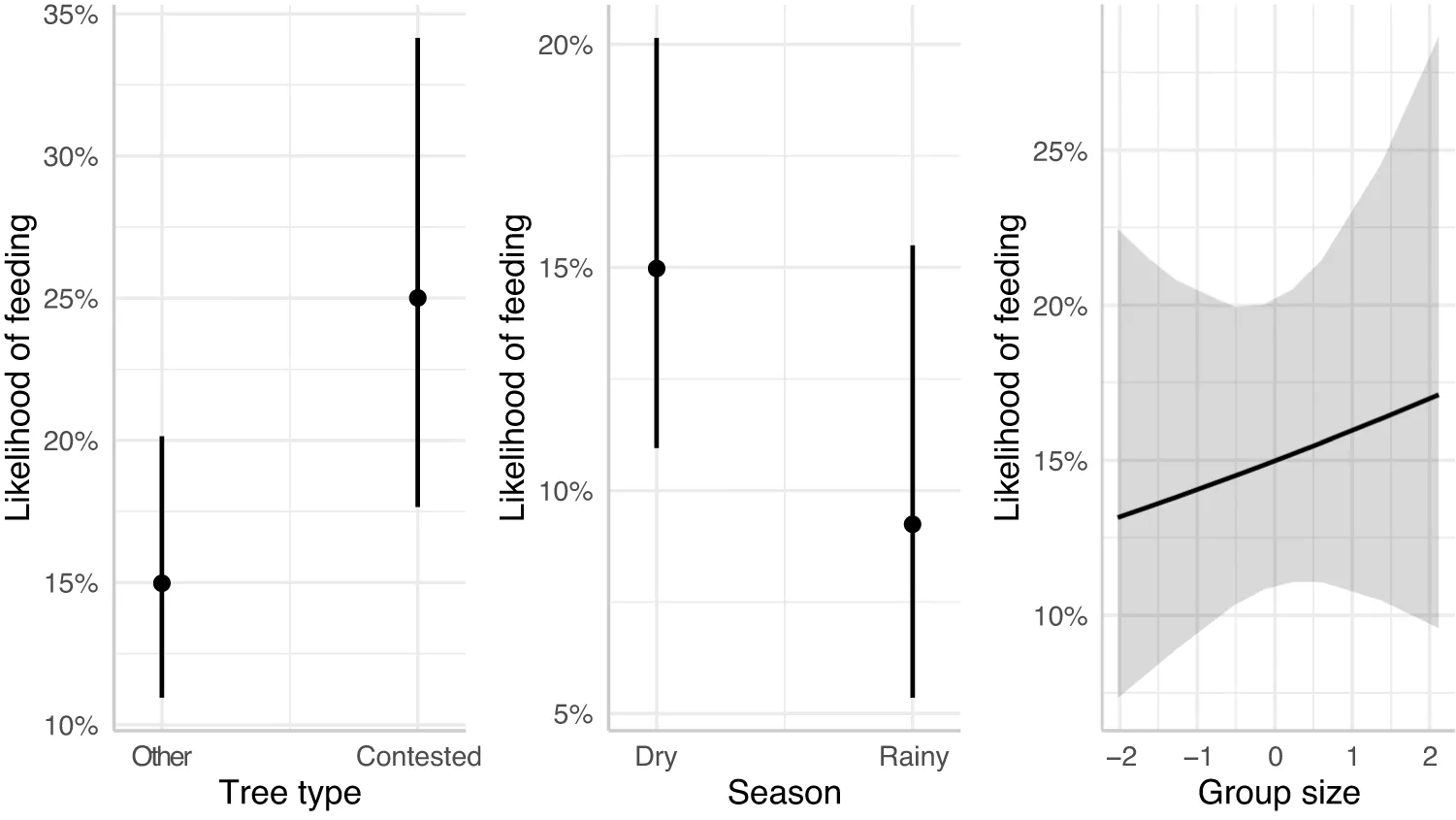
The predicted likelihood of feeding in association with the intergroup encounter based on the tree type (contested tree or other tree), season (dry or rainy), and scaled group size

The likelihood of females feeding in association with intergroup encounters was higher when they were in a contested tree than in other trees (Table 2; Fig. 1). Although the likelihood of feeding in association with intergroups tended to be lower in the rainy season, the 95% confidence interval slightly overlapped zero (Table 2). Group size did not predict feeding in association with intergroup encounters (Table 2). The fixed effects in the model explained 7% of the observed variation in feeding in association with intergroup encounters (R^2^_m_ = 0.07).

**Table 2:** GLMM predicting the likelihood of females feeding during focal follow point samples in association with the intergroup encounter

Fixed effect	Estimate	95% CI
Tree type (contested tree)	0.64	0.22–1.05
Season (rainy)	-0.55	-1.12–0.02
Scaled group size	0.08	-0.19–0.34

The reference levels is “other” for tree type and “dry” for season

### Feeding during the day of an intergroup encounter

**Fig. 2:**
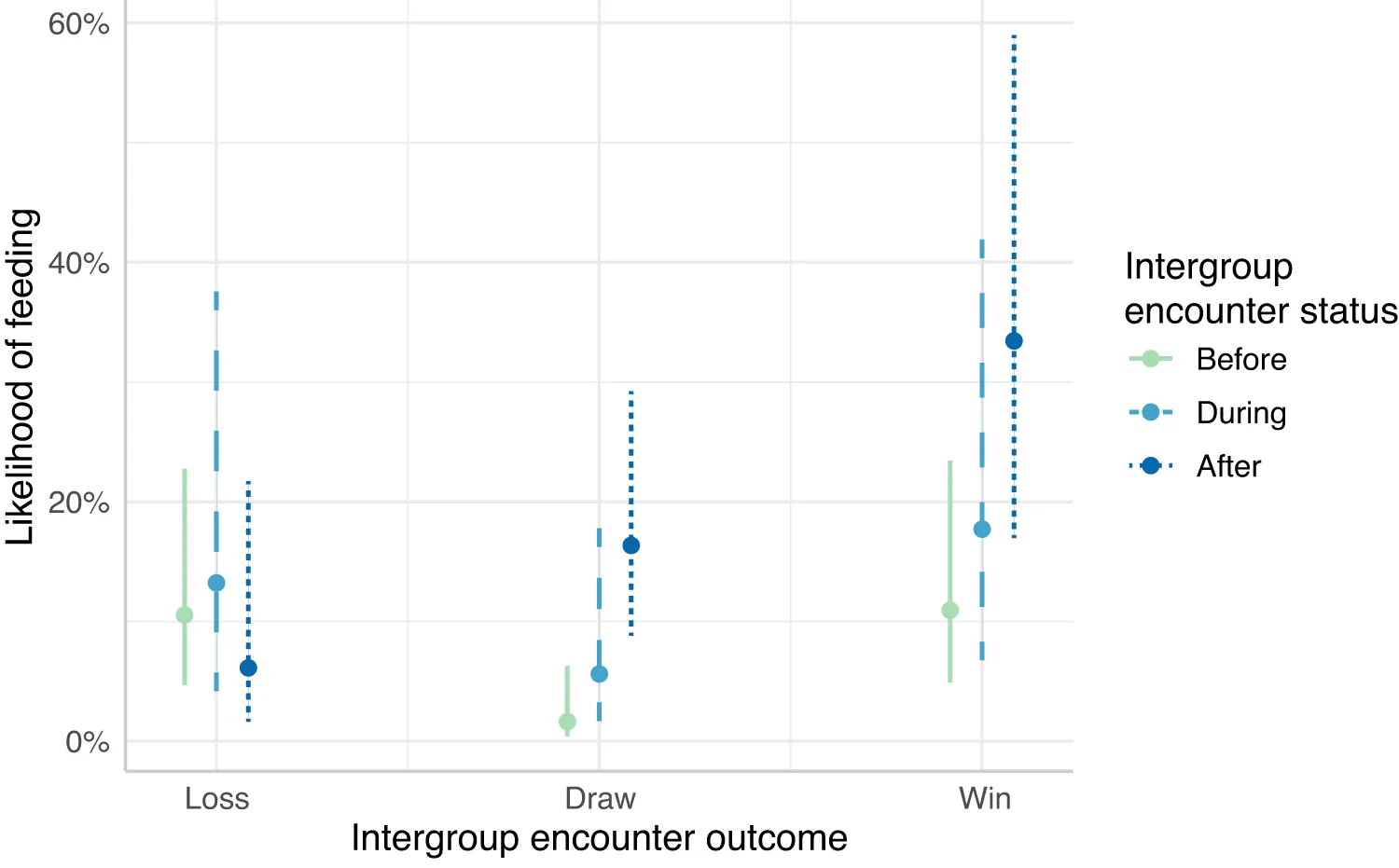
The predicted likelihood of feeding as a function of the intergroup outcome and the intergroup encounter status (solid lines = before, dashed lines = during, and dotted lines = after the intergroup encounter)

During days when the focal group only encountered one opposing group, we collected 637 group scan records of females. Females were feeding in 11% of the group scan records before the intergroup encounter (32/292 individual records up to 8 h before the start), 18% of records during the intergroup encounter (21/117 individual records), and 22% of records after the intergroup encounter (51/228 individual records up to 9 h after the end). The likelihood of foraging in relation to the intergroup encounter status (before, during, or after) differed depending on the outcome (win, draw, loss) (Table 3). The likelihood of foraging before the intergroup encounter was relatively similar across outcomes, while the likelihood of foraging after the intergroup encounter was higher if the intergroup outcome was a win or draw compared to a loss (Fig. 2). The likelihood of foraging during intergroup encounter days increased with group size but was not predicted by season or the interaction effect between group size and outcome (Table 3). The fixed effects explained 35% of the variation in feeding during days with intergroup encounters (R^2^_m_=0.35).

**Table 3:** GLMMs predicting the likelihood of female feeding in group scans during days with intergroup encounters

Fixed effect	Estimate	95% CI
Outcome (draw)	-1.91	-3.38 – -0.44
Outcome (win)	0.04	-1.09–1.16
Intergroup status (during)	0.24	-1.07–1.56
Intergroup status (after)	-0.57	-1.97–0.84
Scaled group size	0.6	0.02–1.18
Season (rainy)	0.63	-0.12–1.37
Outcome (draw) * Intergroup status (during)	1.01	-1.14–3.17
Outcome (win) * Intergroup status (during)	0.28	-1.57–2.13
Outcome (draw) * Intergroup status (after)	2.95	0.99–4.91
Outcome (win) * Intergroup status (after)	1.82	0.07–3.58
Outcome (draw) * Scaled group size	0.34	-0.58–1.26
Outcome (win) * Scaled group size	-0.39	-1.1–0.32

The reference level is “loss” for the intergroup outcome, “before” for intergroup status, and “dry season” for season

## Discussion

During intergroup encounters in the highly folivorous Boabeng-Fiema colobus population when females defended access to specific trees, the contested trees were important food species with high-quality food items present. Females were more likely to feed when they were in contested trees than in other trees during or shortly after the intergroup encounter. The likelihood of feeding on any food resource after the intergroup encounter throughout the remainder of the day was higher if the outcome was a draw or win compared to a loss. These results indicate that winners of intergroup encounters benefit in terms of increased access to important food resources. However, it is still possible that losers can compensate for their loss in other ways, which we discuss further below.

Our analyses focused on intergroup encounters when we could identify specific trees that females were competing over, which made up just over half of the observed intergroup encounters with associated behavioral data. Males did not always show aggression during these intergroup encounters although they participate more than females in intergroup aggression in the full range of intergroup encounters (Arseneau-Robar et al. 2024; Sicotte and MacIntosh 2004; Wikberg et al. 2022a, b). In other aggressive intergroup encounters that do not involve competition over access to a specific tree, the opposing groups may compete for access to a general area of the home range (Sicotte and MacIntosh 2004), especially during the dry season when higher-quality food items are available (Arseneau-Robar et al. 2024). Male-male intergroup aggression is also often due to competition over access to mates in this study population (Sicotte and MacIntosh 2004) and other species (e.g., white-faced capuchins *Cebus imitator*: Perry 1996; Schoof and Jack 2013; mountain gorillas *Gorilla beringei beringei*: Mirville et al. 2018a; Robbins and Sawyer 2007; Sicotte 1993). Males and females in this study population may also direct aggression to extragroup members of the same sex to resist immigration attempts (Sicotte and MacIntosh 2004; Teichroeb et al. 2009) as both sexes disperse (Wikberg et al. 2012). Finally, individuals do not respond aggressively to the presence of other groups in a relatively large proportion of intergroup encounters, with almost half of the observed intergroup encounters lacking female aggression and about a third lacking male aggression (Wikberg et al. 2020). When there is no need to defend resources, non-aggressive intergroup encounters could help to assess dispersal or mating opportunities (Pisor and Surbeck 2019), provide social opportunities (Wikberg unpublished data), and facilitate between-group microbiome transmission (Wikberg et al. 2020). Thus, the function of intergroup interactions can vary across individuals, time, and space.

The perceived value of the contested resource is important in shaping the nature and outcome of intergroup encounters (Maynard Smith and Parker 1976; Parker 1974). Contested trees were of important food species (those that contributed to 5% or more of the annual feeding records) more often than expected based on the distribution of large trees of important food species in the forest. All but one contested tree had young leaves, fruits, and seeds available. The contested trees had young leaves and fruits/seeds more often than expected based on the presence of these plant parts in the monthly phenology surveys. Specific food trees are likely to be more valuable when they have young leaves, although the colobus also eat mature leaves from the same trees year-round (Saj and Sicotte 2007a). During the dry season when many trees have young leaves (Saj and Sicotte 2007a), male and female colobus are more likely to engage in intergroup aggression, which suggests that both males and females defend access to food during intergroup encounters (Arseneau-Robar et al. 2024). Temporal variation in food abundance and competition shapes between-group aggression in many (García et al. 2022; Golabek et al. 2012; Harrison 1983; Korstjens et al. 2005; Lewis et al. 2020; Pal 2015; Thompson et al. 2017) but not all animal populations (Robbins and Sawyer 2007; Thurau and Cords 2024). This between-population variation likely depends on what type of resources individuals in opposing groups compete over. Furthermore, the food characteristics that trigger intergroup aggression vary between populations. For example, intergroup aggression increased with food scarcity in female Verreaux’s sifakas (*Propithecus verreauxi*) (Lewis et al. 2020) and with foraging on seeds from a particular species in female western black-and-white colobus (Korstjens et al. 2005).

Colobus females in this study were more likely to feed on the contested tree than other trees they were in during and immediately after the intergroup encounter. This indicates that contested trees are important trees for feeding in association with the intergroup encounter. Although winners have better access to important food trees during and shortly after the intergroup encounters, colobus monkeys in losing groups would not have to travel far to find another food patch in a different part of their home range as home range sizes are small (Teichroeb and Sicotte 2009). Despite this, the likelihood of feeding on any food resource throughout the day after the intergroup encounter increased if the female’s group won or drew in comparison to if they lost. The likelihood of feeding was similar before the intergroup encounter regardless of its outcome. Our results indicate that there are food-related benefits of winning intergroup encounters in this study population. Individuals in losing groups may not be able to make up for losing access to contested food trees by feeding more before the intergroup encounter or on different resources after the intergroup encounter.

In other studies, the outcome of the intergroup encounter has been associated with ranging patterns that further point to immediate or more long-term costs associated with losing the encounter, including increased travel distances and/or reduced use of the area during the following months (Crofoot 2013; Koch et al. 2016; Markham et al. 2012). However, losing intergroup encounters is not always associated with foraging (Harrison 1983) or energetic costs (Brown et al. 2022). Furthermore, individuals in groups with low competitive ability may avoid intergroup encounters by establishing home ranges further away from the core of the forest where home range overlap is highest. Two study groups in the years prior to and including this study period increasingly ranged in the secondary forest along the edge of the study area as they lost parts of their home ranges close to the core forest to newly established groups, including fission products (Wikberg, unpublished data). These groups are now involved in fewer intergroup encounters than other groups in this population (Wikberg et al. 2022a, b). Although the core of the study area contains more primary forest, the behavioral flexibility exhibited by this species (Wong and Sicotte 2006) and by some other African colobines (reviewed in Wikberg et al. 2022b) may enable them to maintain high energetic status and reproductive output in different habitat types. It is therefore important that future studies include physiological measurements (Brown et al. 2022; Lucchesi et al. 2020; Schoof and Jack 2013) or data on reproductive output (Cheney and Seyfarth 1987; Lemoine et al. 2020a, b; Thompson et al. 2017) to understand the long-term consequences of intergroup encounters for individuals in winning and losing groups.

## Data Availability

The data used for the analyses presented are available on figshare https://doi.org/10.6084/m9.figshare.29104229.v1.
